# On the Utility of Large Doses of Opium

**Published:** 1818-06

**Authors:** Nicholas Littleton


*For the London Medical and Physical Journal.*

### *On the Utility of large Doses of Opium;* by NICHOLAS LITTLETON, Esq.

AN error, either from unwarranted boldness, or indecisive timid practice, seems equally unfortunate, as both may be equally fatal: on each side of the path may be an abyss.—Error, how infinitely more attainable than truth! Now, as navigators very properly make known sunken rocks and safe harbours by their charts, so all observers ought to publish whatever they may suppose new, or not generally known, especially in such a profession as is the art of healing, where so much may be done by the timely application of even trifles.

Many may think the use of opium sufficiently known, as it has been so long in use, so variously used, and by such varied characters of medical practitioners. But there are many points undecided, though long discussed; many questions unanswered, though long proposed.

The use of opium, though so old as the time of Hippocrates, was then used in a very trifling manner, in very small quantities, and on very few occasions.—All the ancient Greek and Roman writers seem to have used it very undecidedly. They had their jargons of Mithridate, Theriaca, &c. leaving us to guess whether they really considered the efficacy of these remedies to depend on the mixture, or on a single article entering into such compositions. Paracelsus, I believe, first introduced a solution of it as a remedy, with the captivating name Laudanum, *(an a Laudandum).* But it is to the moderns that we are chiefly indebted for the most useful observations on opium.

One of the oldest modern authors that I know of, who treats on the use of opium, is the truly celebrated Sydenham. The writings of this great physician are so grounded on observation, that his directions have been commonly followed, even down to the present day; at least, I believe it is generally the case with respect to the use of opium, on which he has made many valuable observations, particularly directing the dose to be given according to the violence of the symptoms and the nature of the disease. When writing on bilious colic, page 165 of Dr. Pechy's translation, he says, "Nor could I ever take off violent pains without a larger dose than is usual, and that repeated too; for that which is sufficient to conquer another disease will be wholly insufficient in this case,—the violence of the disease subduing the force of the medicine; and it is, indeed, safe to repeat the narcotic according to the degree of the pain, till it ceases, or is very much lessened; yet, there must be such a space of time between them, that I may find what may be hoped for from the former dose before I give another." And, again, pages 358 and 359, "The repetition of it must be according to the greatness of the symptoms,—for that dose which will be sufficient to quell a small symptom, will be overcome by a stronger; and that which would otherwise *endanger the life* of the patient, will, in such a case, save his life." He gives his largest doses in cases of cholera, bilious colic, and hysteria, but seems never to have ventured on larger doses than a grain and a half of solid opium, or twenty-five drops of an infusion of a drachm of opium in an ounce of wine, and these doses were repeated at very distant intervals. Such small doses, and so repeated, I am convinced would have produced no effect whatever in cases similar to those I shall hereafter record. Nevertheless, Sydenham seems to have expected, that the charge of boldness would be raised against him. "Nor is there any reason why any one should count me too bold, because I venture to give so great a quantity of liquid laudanum." Astonishment may arise in the minds of some readers at the largeness of the doses which have been given in the following cases, but such readers should recollect, that astonishment arises from inexperience; they should, therefore, suspend their judgment till opportunity enables them to judge correctly. I am, therefore, (not perhaps without sufficient reason,) careless of such condemnations as may arise in the minds of any, merely from such astonishment.

One of the oldest writers that I know of, who has directed what may properly be called large doses of opium, is Dr. Dovar, who, page 12 of the Physician's Legacy, has the following prescription to ease the gout:—Take, of opium, one ounce; salt-petre, and vitriolated tartar, of each four ounces; ipecacuanha, one ounce; liquorice, one ounce. The salts are to be exposed to a red heat as long as flames arise; afterwards, to be powdered with the opium; and, finally, all are to be mixed together. The dose he directs of this powder is forty to seventy grains in a glass of wine at bed time, which may be equivalent to from four to six grains

2

of opium. He remarks, that in two or three hours, at furthest, the patient will be perfectly free of pain. I should have no doubt that opium might, with perfect safety, be given in a quantity sufficient to alleviate completely the distressing pain of gout; inasmuch, as pain in gout seems to depend on a particular constitution of nerve, different from the state prevalent in common inflammation. The old treatment, however, of this latter disease, seems as perfect as can be hoped for; at least, such is my conviction, arising from the great success I have seen attendant on copious bleeding, that I shall never be induced to administer opium in order to remove the pain of common inflammation, as its delusive relief might lead to the destruction of a life otherwise easily preserved. I should not have thought of making these last remarks on opium, had I not read the writings of an M. D., where opium was recommended in largish doses for common inflammation, and even venesection condemned in every case, whether of disease or injury, as being never necessary and seldom safe.

I have read with pleasure, a case, where the surgeon acted on the very principle which seems so much deserving of notice, and which it is the intention of this paper to particularly recommend. The case is stated by Mr. Brandish, of Alcester, in the London Med. and Phys. Journal for 1786, vol. vii. page 136. It is of a boy, æt. 14, who was found to require four to six drachms of the tincture of opium to relieve the torture caused by an urinary calculus. I have, in my reading, met with many such detached cases of the utility of large doses of opium, and, no doubt, I might find many more by turning over the various medical writings; but all such quotations of cases would be only filling the pages of your valuable Journal with too much unnecessary matter. And, as cases, observed by myself, bring most conviction to my mind, I shall chiefly rely on the statement of these, in order to induce others to adopt a practice which I can depend will produce self-conviction.

But, as these self-convinced may, notwithstanding, consider him who first ventured to recommend such poisonings, as unwarrantably bold, I shall, therefore, relate my progress to this decisive practice.

My teachers, it is true, never recommended more than gtt. 30 to gtt. 60 of the tincture, or two to four grains of solid opium, excepting in tetanus, hydrophobia, and in some violent cases of cholera; also, medical writers in general, gave similar directions. From a few, however, passages were to be read which conveyed different ideas; but no one seems to have dwelt so much on the use of large doses as Darwin: this practice happily according with the theory which pervades his Zoonomia. His theories I considered as deserving little regard; but the statement of such valuable facts as are scattered through the whole of his work, and especially those on the administration of opium, (vide vol. iii.) left indelible impressions on my mind, which chiefly led me to venture on doses of half a drachm of the tincture in the following case.

About two years ago, the wife of N. H., æt. about 30, was suddenly seized with violent pain at the scrobiculus cordis, extending to the left side so as almost to deprive her of the use of her senses: she was writhing the body in the greatest agony, undoubtedly arising from spasm of the stomach. I directly gave her half a drachm of the tincture, and was obliged to repeat it twice before the pain ceased, which it did almost instantly on giving the third dose. Some weeks after, she was again attacked in the same manner, when I gave a drachm at one dose, as before I had found it necessary to give a drachm and a half: this increased dose, in a few minutes, rendered her perfectly easy. Some time after this, she was again attacked much more violently than ever, when it was necessary to give, and even to repeat, the drachm-dose. I had, before this time, remarked, that, when relief was consequent to the administration of opium, it took place within a quarter of an hour; and I have ever found it to be so.—Whence patients, admitting of relief from opium, may escape much suffering, and even danger, by giving the tincture more frequently than Darwin and others mention: thus, also, less opium will be found necessary than when the dose is only repeated every half-hour or hour. After such large doses, sleep was not always to be expected; sometimes she did not sleep at all, at other times she did, though not soundly; and always, in four to eight hours after the relief from pain, sickness occurred, which might or might not subside in an hour or two, so as to allow food to remain on the stomach. Constipation does not always follow such large doses, but may be sometimes expected. In this female, no cathartic remedy was found necessary after such quantities of opium. She had no attack of pain afterwards, whilst she remained in Padstow, which was a period of some months; and, I have since been told by her husband, that she has had no return, and that she enjoys good health. In this case, it is not difficult to divine what would have been the effect of the tincture of opium, in doses of thirty drops repeated every half hour, as recommended by Sydenham, and as, I believe, is now the common practice. I have heard a surgeon speak, rather with wonder, of a lady who took 100 or 200 drops through the whole course of a night, in doses of thirty drops, in an affection similar to that which I have just related. The dreadful sufferings to be escaped by a more decided practice, are quite sufficient to determine for ever the manner of proceeding in such cases.

Miss P——, æt. 30, had been subject to spasms of the stomach for several years, so as to have been confined for several months about two years since. During the night-time, she was attacked with the violent pain, which she well knew. When I arrived, I gave between half a drachm and a drachm of the tincture, by which the pain soon ceased. This dose caused vomiting, but neither sleep nor constipation.

Mrs. R——, æt. 25, rather delicate, had been subject to convulsions for many years, especially after frights or violent pains, as of the bowels, &c.: in her attacks, having had convulsions returning at short intervals for days together. During the night-time, she was seized with convulsions, which lasted from five to ten minutes: she again felt the uneasy sensation usually preceding the convulsion; but, on giving half a drachm of the tincture, it ceased. She had quietude, but no sleep, and the tincture caused vomiting of every thing for twelve hours after the commencement of the sickness. Some time after, she had much pain in the abdomen, such as often preceded, or sometimes seemed to cause, the convulsions: she had a mixture, containing opium, and had taken a drachm of the tincture, but, notwithstanding the presence of so much opium in the stomach, she had an attack of convulsion, but not of long duration:—Is it not likely that a larger dose would have prevented it?

A. M. æt. about 40, on hearing it reported that her husband was drowned, became distracted and convulsed. During a short cessation of convulsion, I succeeded in getting her to swallow a drachm and a half of the tincture. The convulsion did not return, and she remained calm, having neither sleep, vomiting, nor constipation, succeeding the dose.—Fortunately, her mind received the greatest consolation not long afterwards,—that of hearing the report was false.

E. M., æt. 36; seven or eight months pregnant, heard the dreadful intelligence of her husband's drowning. She became distracted. I saw her soon after: she had not been convulsed: gave her one drachm of the tincture, and repeated it in ten or fifteen minutes: she became calm, and remained in silent sorrow, free from loud shriekings, or violent muscular exertions: in about four hours, she vomited, and the stomach did not retain ingesta for six or eight hours. No sleep or constipation followed. She continued in silent distress for some time, which gradually abated, and she was delivered of a healthy child six or eight weeks after.

S. W. æt. 42; found her in the greatest mental distress, she having just heard of her husband's drowning: she was often convulsed. As she was rather of a strong constitution, I bled her to ℥xv., and then gave between two and three drachms of the tincture, at two doses. She afterwards became tolerably composed, and gradually recovered from the shock.

Mr. F——, æt. 50, of a gross full habit, but temperate in his manner of living. About twelve months ago, he suffered from a carbuncle between the scapulæ, not having applied for assistance until it had extended to nearly six inches in diameter: he has also had several biles since, on different parts of the body, of a carbuncular nature: he is often troubled with relaxed bowels. Whilst in the field, he was seized with violent pain in the abdomen; he returned home, and went to bed, often groaning from the severity of the pain. I saw him about two hours afterwards: he had been suffering, at short intervals, the same excruciating pain, attended with frequent vomiting: the bowels were not constipated, nor was there any pain caused by pressing on the abdomen. I immediately gave one drachm of the tincture, and, as the pain returned slightly, I repeated it in fifteen minutes; and ordered, if the pain should return, to give a small mixture, containing a drachm and a half of the tincture, at two doses every fifteen minutes, till the pain ceased. The pain returned about four or five hours after requiring the whole of the mixture to be given. Afterwards, he had no return of pain, and only required a strong cathartic on the next day, being soon quite recovered.

Mrs. H., æt. 38, tall and delicate, was seized with violent pain in the abdomen: she could not bear pressure, as the parietes abdominis were so tender, and, whenever the pain increased in violence, she felt as if the parieties were drawn together, and an inclination to void fæces. I gave half a drachm of the tincture, and repeated it in ten minutes; but, as she still continued distracted with pain, I bled her to faintness at ℥xv.: during the faintness, the pain was relieved. An enema could not be injected; a purge was therefore given, but still the pain did not seem quite gone, although she continued tolerably easy for two days, when the pain returned as violent as ever. I then gave her the tincture in drachm-doses, and, after repeating it once or twice, the pain entirely ceased. This occurred about six months ago, since which time she has enjoyed her usual health.

B.B., æt. 19, a hardy robust girl, had a little pain in the bowels through the day; had an alvine evacuation in the forenoon: in the evening, she was seized with violent pain between the scrobiculus cordis and umbilicus, on which part she could not bear pressure: she lay with her body bent forwards, often shrieking with pain. She said, that, when the pain first attacked her, leaning with the painful part against the edge of the table gave relief. I gave about two drachms and a half of the tincture in fifteen minutes: the pain still continuing, I bled her to fainting, when the pain became relieved; but, as it soon returned, I again allowed the arm to bleed, so that, altogether, she lost above ℥xxv. of blood. Waiting about a quarter of an hour, the pain again returned, I, therefore, gave a drachm and a half of the tincture, by which she became easy. The whole of the opium was taken in about an hour and a half, or two hours time: four or five hours after, she vomited. The following morning, took a few cathartic pills, and required nothing more.

E. W., æt. 19, a hardy robust girl; had felt pain in the abdomen all the morning, which had sometimes been so violent as to occasion crying. About the middle of the day, she became distracted with pain: she lay with her body bent together, and could not bear pressure: the pain was of a cutting nature: the bowels not constipated. I gave about half an ounce of the tincture in twenty minutes. As this did not relieve, I bled her to faintness, when she became tolerably easy. I remarked that she was fully under the influence of opium, as the pupils were small, although she lay with her eyes open and exposed to but little light, answering many questions that were asked her. In the evening the pain returned: I again allowed the arm to bleed, so that in all she lost ℥xxx., or more, of blood: the pain still continuing, I gave a drachm, or more, of the tincture,—by which she became easy. So much opium caused vomiting, but no sleep, and she only required a powerful cathartic afterwards.

Miss S——, æt. 29,—Tuesday, February the 26th, 1818,—was seized with pain in the evening, which continued throughout the night and following day. The Wednesday night she slept tolerably well; but, on the Thursday, the pain was equally severe. In the evening, about nine o'clock, I first saw her: she complained of violent pain at, or a little above, the umbilicus: she could not bear pressure on that part of the abdomen: she felt a constant dull aching, with severe stabbing shooting pains: she has had no fæcal evacuation since Tuesday. I immediately bled her, she being strong and robust, to ℥xxx., or more, which caused faintness: the dull aching still continued, but the stabbing was less severe. I then gave about a drachm and a half of the tincture, when, in about ten minutes, she became quite easy, so that she would hardly consent to have an enema injected; I, however, directed a common enema to be injected every two hours, till a fæcal evacuation followed; also ordered a powder of calomel, gtt. viii. and two large meat-spoonsful of a saturated solution of the sulphate of magnesia, to be given every two or three hours till the bowels were certainly acted on. The next morning, I found she had had six or seven evacuations, containing scybala: she vomited at about two or three o'clock in the morning, and still complains of nausea. She has had no pain since last night, and feels only slight soreness on pressure. She is thirsty, as is evident from the frothy saliva on the edges of the tongue: such thirst is very common after large doses of opium. She took nothing worth mentioning after, and soon recovered.

Mrs. Y., æt. 35, has had three children: always after delivery, could not sleep for three or four nights, although composing draughts were given, and she was perfectly free from pain. By giving between half a drachm and a drachm of the tincture, she repeatedly slept well. It did not cause vomiting or constipation.

If this communication should prove acceptable, at a future time additional cases will be given, as these are but a few of all I have observed, where opium has been given even in much larger quantities than any I have as yet mentioned.

*Padstow; March* 7, 1818.

